# Intraoperative arthrography favorably impacts the early outcome of operatively managed fractures of the lateral humeral condyle displaced 1–5 mm in children

**DOI:** 10.1186/s13018-022-03472-z

**Published:** 2022-12-27

**Authors:** Ali Lari, Ahmad Alenezi, Jarrah Abughaith, Haitham AlShehawy, Wael Hammady, Saleh AlSaifi

**Affiliations:** Department of Orthopedic Surgery, AlRazi Orthopedic Hospital, Kuwait City, Kuwait

**Keywords:** Lateral humeral condyle, Pediatric trauma, Arthrogram, Operative, Percutaneous pinning, Radiography, Minimally displaced

## Abstract

**Background:**

Controversy exists surrounding the optimal approach to managing pediatric lateral humeral condyle fractures (LHCF). The difficulty in assessing the articular surface using radiography and the intra-articular element potentially involved make LHCF susceptible to complications and delayed diagnoses. Arthrography has been used to delineate the articular surface to aid in deciding whether closed or open reduction is necessary. However, there has been scarce evidence to determine the accuracy of using radiography versus arthrography to predict articular disruption in LHCF displaced 1–5 mm. This study assesses; (1) the utility of intraoperative arthrography in modifying the method of operative reduction, (2) the accuracy of plain radiography in identifying articular integrity, and (3) the clinical outcomes of early operative treatment.

**Methods:**

This was a single-center prospective study that involved operatively treated pediatric LHCF with a displacement of 1–5 mm. Patient demographics, radiographic displacement, predicted radiographic articular integrity, articular integrity on arthrograms, modification of management and follow-up clinical outcomes were obtained.

**Results:**

A total of 72 patients were included with a mean displacement of 2.6 mm and a mean follow-up of 16 months. The articular surface was disrupted in 21% of patients. The reduction method (open versus closed) was modified in 15 patients (21%) after an intraoperative arthrogram. Out of 25 patients with displacement < 2 mm, four of which (15%) had disrupted articular surface and were subsequently treated with open reduction internal fixation (ORIF). While eleven patients with > 4 mm displacement had an intact articular hinge that were managed with closed reduction and percutaneous pinning (CRPP). All patients achieved union with no documented major complications. The ability of radiography to discriminate between disrupted/ intact articular integrity decreases as displacement decreases.

**Conclusions:**

Data from this study suggest using the degree of displacement measured on plain radiography is insufficient in predicting articular integrity for fractures displaced 1–5 mm. The use of arthrography guides reduction method and adequacy, avoiding scenarios of unnecessary open reduction and insufficient closed reduction. Further, a significant amount of outliers exist that have intact articular hinges above 4 mm and disrupted hinges below 2 mm of displacement. Finally we report favorable outcomes using a lower threshold for early operative treatment.

## Introduction

Pediatric lateral humeral condyle fractures (LHCF) represent 10–20% of elbow fractures in the pediatric population [1, 2]. Despite their ubiquity in pediatric trauma, the optimal management of this fracture remains controversial, as indicated by growing evidence and shifting paradigms. The intra-articular element of the fracture and the difficulty to accurately assess it with plain radiographs make lateral condyle fractures prone to complications and delayed diagnoses [3–5].

Surgeons are often faced with several challenges. While the diagnosis is often made by plain radiography, much of the distal humerus is unossified in young patients and is radiolucent. This may confound the accurate determination of the degree of displacement [6]. Further, the integrity of the articular cartilaginous hinge and the degree of intra-articular displacement have been considered equally important in determining stability [7]. Classifications, including that described by Weiss et al. have been developed to guide management based on the integrity of the cartilaginous hinge [8]. However, classification systems have been limited in their application due to the variability of radiographic assessment, as well the inter-observer variability [4, 6, 9]. This is of particular importance as a difference of 1–2 mm may considerably alter management.

Despite a plethora of evidence on the management of LHCF, definitions of displaced and minimally displaced fractures remain unclear. Fractures that are displaced < 2 mm have classically been management conservatively, whereas those displaced > 2 mm have been managed operatively on the basis of indeterminable stability [3, 5, 10, 11]. It is likely that decision-making is affected by the increased rates of complications associated with fractures that have displaced after initial conservative management [5, 11].

Further, there is growing evidence that has demonstrated a considerably higher rate of failure in conservatively managing fractures displaced > 1.2 mm, further advocating for early intervention in LHF displaced < 2 mm [12]. In addition, while Weiss et al. utilized the arthrogram to assess fractures displaced 2–4 mm, there is a paucity of data on the utility of this technique in fractures displaced < 2 mm and > 4 mm.

On this basis, the purpose of this prospective study is to; (1) analyze the utility of the intraoperative arthrogram in modifying the method of operative management in comparison to plain radiography for fractures displaced 1–5 mm, (2) describe the accuracy of the radiograph in discriminating between intact and disrupted articular surfaces, and (3) report the clinical outcomes of early operative treatment.

## Methodology

### Study design and study population

This was a single-center, prospective study that was carried out after obtaining approval from the institutional review board. Informed consent was obtained from eligible patients and their parents prior to their inclusion in the study.

Between February 2018 and March 2021, eligible patients were consecutively enrolled if they were below 12 years of age and presented within 24 h of injury with an isolated humeral lateral condyle fracture that was displaced between 1 and 5 mm. Patients were followed up for a minimum of one year. Exclusion criteria entailed; open fractures, ipsilateral extremity fractures, metabolic bone disease and severe soft tissue swelling.

### Outcome assessments

Patients were evaluated according to age, sex, side of injury, displacement on plain radiographs, Song classification, pre-arthrogram radiographic plan based on the provider’s prediction of articular surface status, presence of articular disruption on arthrogram and post-arthrogram plan. Further, patients were assessed throughout the postoperative course for; follow-up period, range of motion (ROM), complications, lateral spurring, fishtail deformity and Hardacres criteria. On a calibrated, computerized system, the displacement was measured using the greatest distance between the proximal fragment and the distal fracture fragment on the internal oblique view by the consensus of two trained pediatric orthopedic surgeons. This was usually the displacement measured between the lateral cortices.

### Treatment protocol

Patients were initially assessed in the emergency department of a tertiary orthopedic hospital. After initial physical examination, patients were placed in an above elbow slab, admitted and booked for surgery. The treatment plan consisted of either closed reduction and percutaneous pinning (CRPP) or open reduction and internal fixation (ORIF) with Kirschner wires. The pre-arthrogram plan was initially based off the internal oblique views. The post-arthrogram plan occurred intra-operatively based on the congruence of the articular surface.

**Fig. 1:**
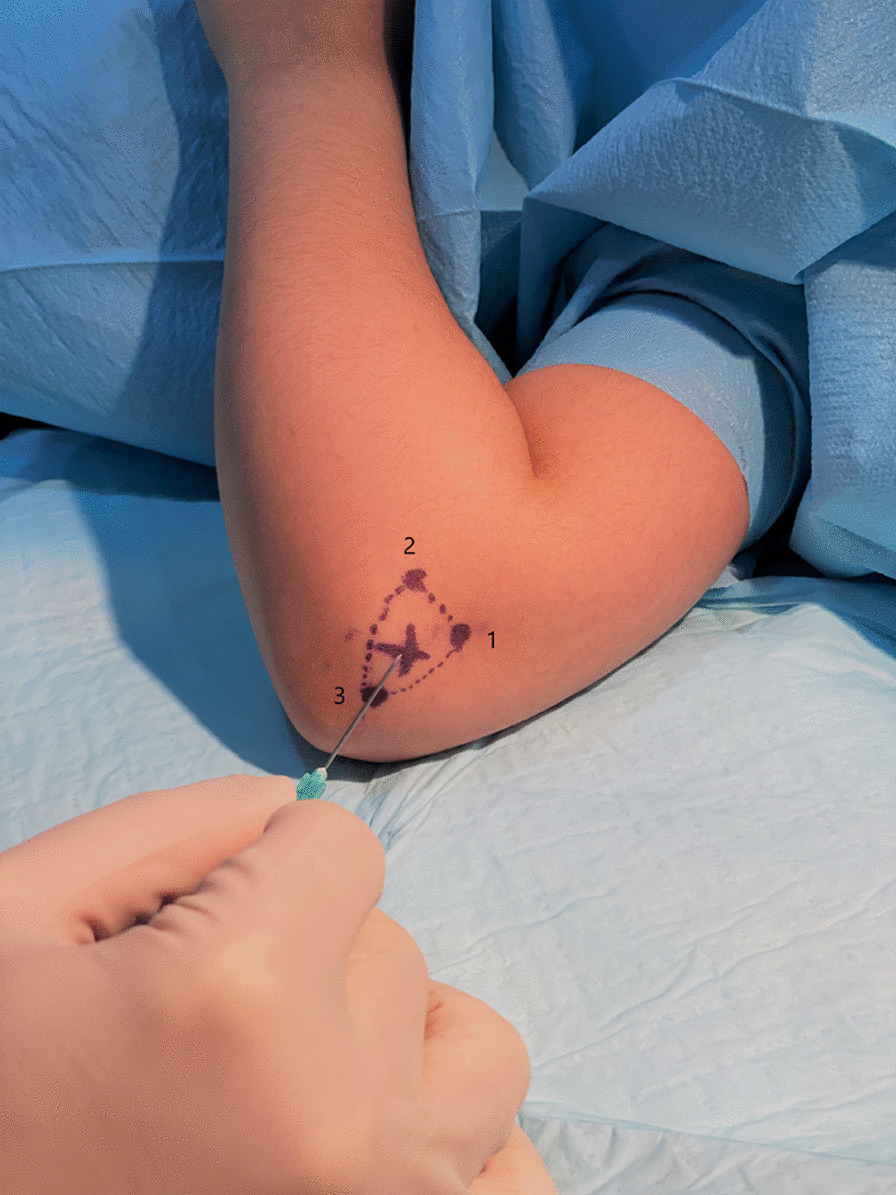
A photograph of the triangle that delineates the “soft spot” of the elbow used to inject dye into the articular space. Borders include 1; lateral epicondyle, 2; radial head, 3; edge of the olecranon

Under general anesthesia and aseptic technique an elbow arthrogram was performed in the operating room. Using a 20–22 gauge needle, arthrography was performed by injecting contrast into the elbow joint by utilizing the lateral approach. This was performed by injecting the dye into the “soft spot” of the elbow joint by using a drawn triangle made by the following landmarks; the lateral epicondyle, radial head and the olecranon (Fig. 1). Next, the needle is inserted anteromedially at a 45° angle. The needle’s position is confirmed under fluoroscopy to avoid injecting into the surrounding soft tissue and obscuring vision.

Fluoroscopic images were obtained to delineate the articular cartilage of the humerus. The decision to perform CRPP versus ORIF was dependent on the integrity of the articular cartilaginous hinge. Closed reduction and percutaneous pinning was performed if the articular surface was intact. Kirschner wires were placed as perpendicular to the fracture as possible. A total of two to three wires were used in a crossing and divergent configuration. Additionally the wire was sometimes placed transversely just above the joint line.

Open reduction and pinning was performed if the articular surface was disrupted and dye was seen in the articular space. The decision was dependent on the presence of dye in the space as intraoperative assessment of displacement using fluoroscopy is technically and logistically difficult. Further, in cases where dye is seen trickling into the fracture site but the articular surface is still smooth and well delineated, open reduction was not performed. This was considered an intact articular hinge.

In the postoperative course, patients were immobilized in an above elbow slab for two weeks. Next, patients were assessed weekly in the outpatient department (OPD). Kirschner wires were removed at approximately 4 weeks postoperatively. Depending on the radiological evidence of healing and resolution of fracture site tenderness, patients were allowed to mobilize.

### Statistical analysis

Statistical analysis was performed using R v 3.6.3. Counts and percentages were used to summarize the distribution of categorical variables. The mean ± standard deviation (SD) and the median/interquartile range [IQR] were used to summarize the distribution of continuous normal and non-normal variables, respectively. Binary logistic regression to construct a model that can be used to predict the presence of a disrupted articular surface. Model fit was assessed using sensitivity, specificity, PPV, NPV and accuracy. Bootstrapping using 1000 bootstrapped samples was used to validate the model.

## Results

Between February 2018 and March 2021, a total of 85 patients were diagnosed with LHCF. Seven patients were excluded for having displacement < 1 mm. Four patients were excluded for loss of follow-up. Two patients were excluded for refusing initial operative management. A total of 72 patients met the inclusion criteria and were consecutively recruited into the study (Table 1).

**Table 1:** Descriptive statistics for the study group

	[Group] *N* = 72
*Sex*	
F	24 (33%)
M	48 (67%)
*Age*	
Mean (SD)	5.67 (2.23)
*Side*	
Left	32 (44.4%)
Right	40 (55.6%)
*Song classification*	
2	16 (22.2%)
3	17 (23.6%)
4	31 (43.1%)
5	8 (11.1%)
*Displacement*	
Mean (SD)	2.63 (1.05)
*Displacement CRPP to ORIF (N* = *7)*	
Mean (SD)	2.07 (0.51)
*Displacement ORIF to CRPP (N* = *8)*	
Mean (SD)	3.89 (0.55)
*Articular surface*	
Disrupted	15 (20.8%)
Intact	57 (77.8%)
*Pre-arthrogram plan*	
CRPP	56 (77.8%)
ORIF	16 (22.2%)
*Post-arthrogram plan*	
CRPP	57 (79.2%)
ORIF	15 (20.8%)
*Plan modified*	
No	57 (79%)
Yes	15 (21%)
*Follow-up period*	
Mean (range)	16 months (12–24)
*Range of motion*	
Full	68 (94.4%)
Limited	4 (5.56%)
*Lateral spurring*	
No	36 (50.0%)
Yes	36 (50.0%)
*Fishtail deformity*	
No	70 (97.2%)
Yes	2 (2.78%)
*Hardacres criteria*	
Excellent	66 (91.7%)
Good	6 (8.33%)

CRPP, closed reduction and percutaneous pinning; ORIF, open reduction and internal fixation

The average age of the included patients was 5.67 ± 2.23 years. The average displacement was 2.63 ± 1.05. The articular surface was disrupted in 21% of the patients and intact in the remaining 79%. The reduction method (Open versus closed) was modified in 15 (21%) patients after intraoperative arthrogram assessment.

**Fig. 2:**
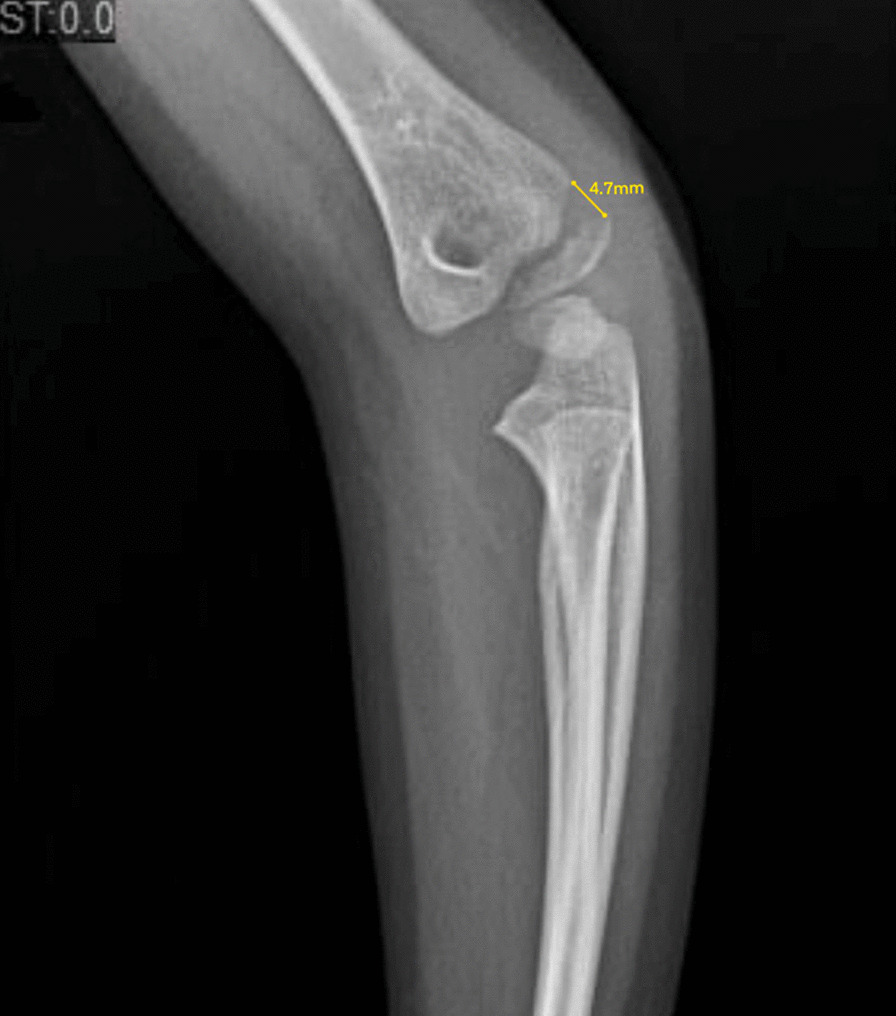
Radiograph demonstrating a fracture of the lateral humeral condyle with a measurement technique showing a displacement of 4.7 mm

**Fig. 3:**
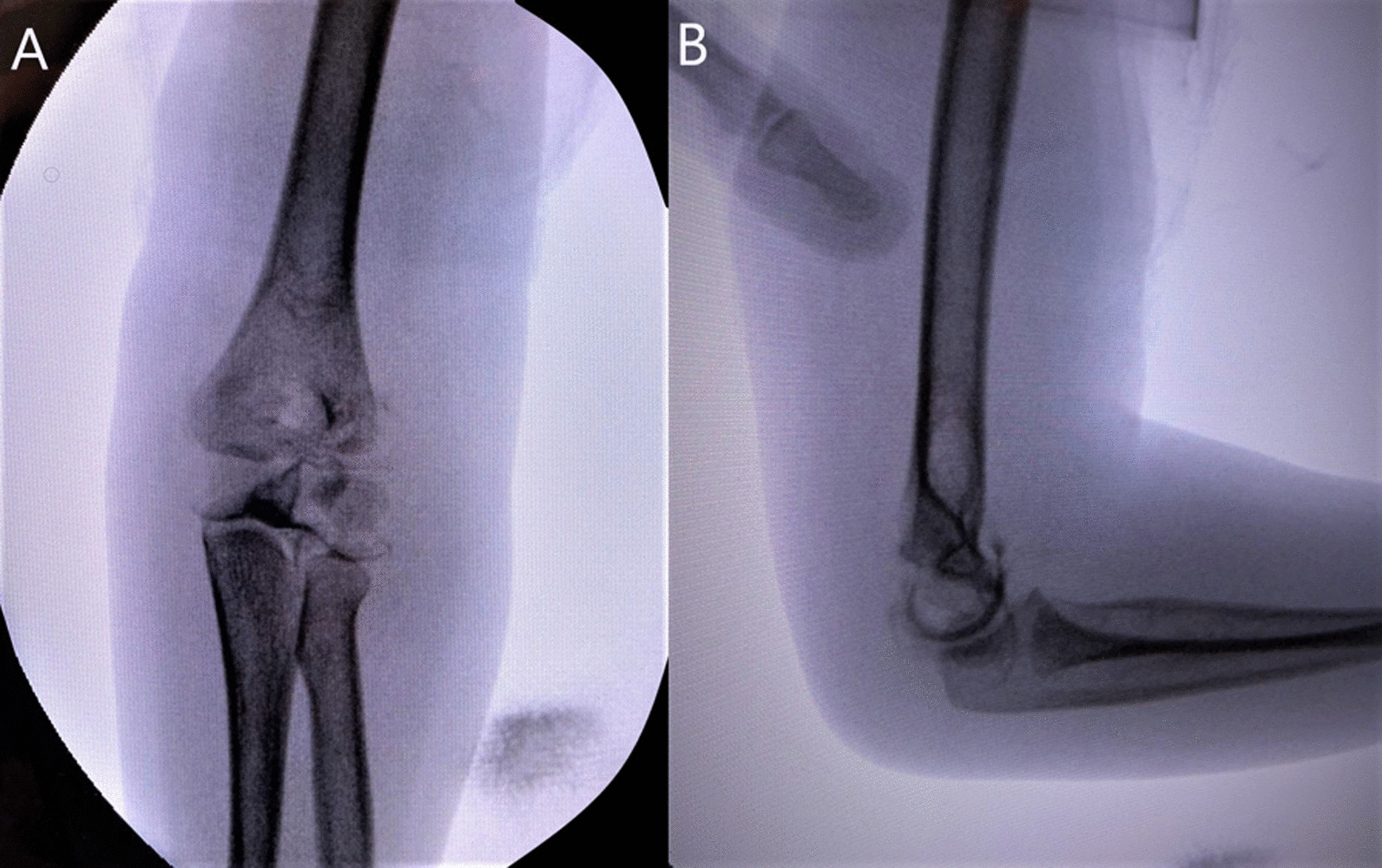
**A** An AP view of the same patient’s intraoperative arthrogram revealing an intact articular hinge. **B** A lateral view of the intraoperative arthrogram revealing an intact articular hinge

Overall, 25 patients had displacement < 2 mm, four of which (15%) revealed a disrupted articular surface during arthrography (range 1.5–1.9 mm). A total of 36 patients had displacement between 2 and 3.9 mm, six (16.6%) of these patients had their plan modified after assessment with an arthrogram. Eleven patients had a displacement of 4–5 mm, of which five of these patients (45%) revealed an intact articular surface and were subsequently treated with CRPP (range 4–4.7 mm). An example of a LHCF displaced 4.7 mm is displayed in Fig. 2. This case was initially predicted to have a disrupted articular hinge. An intraoperative arthrogram revealed an intact articular hinge on anteroposterior and lateral fluoroscopic images (Fig. 3). This patient was subsequently treated with closed reduction and percutaneous pinning.

**Fig. 4:**
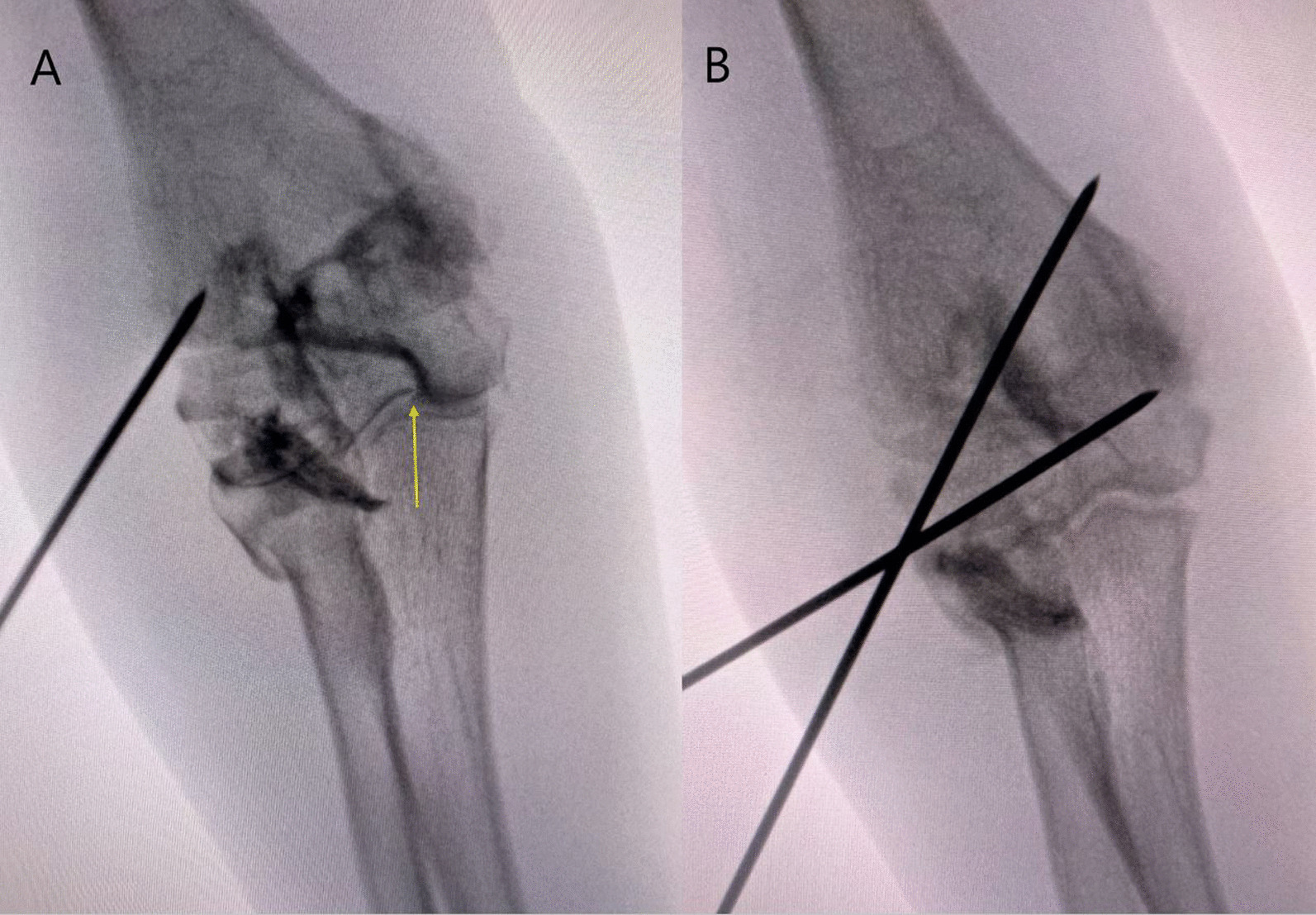
**A** Intraoperative arthrogram of a minimally displaced LHCF revealing a disrupted articular hinge with dye leaking into the joint through the fracture (yellow arrow). **B** Immediate postoperative fluoroscopic image after open reduction and pinning with a congruent articular surface

On the other hand, an example of a LHCF displaced 1.9 mm initially meant to undergo closed reduction and pinning was found to have a disrupted articular hinge (Fig. 4). This patient underwent open reduction revealing a disrupted articular hinge with a slightly rotated fragment.

All of the patients achieved union. There were no documented cases of; infections, angular deformities, avascular necrosis and malunion. Four out of the 72 patients had 10 degrees of extension deficit at the one-year follow-up. All four of the patients underwent open reduction and pinning for a disrupted articular hinge.

Although there was a clear gradient between displacement and disruption (Table 2), a cutoff of 2 mm displacement was only 75% sensitive to predicting articular disruption. The data suggest that the predictive ability of displacement to discriminate between intact and disrupted articular integrity increases as displacement increases.

**Table 2:** Predictive ability of displacement to discriminate intact and disrupted articular surface

Cutoff	Accuracy	Sensitivity	Specificity	PPV	NPV
*Displacement*					
≥ 1.5	31.94	100	12.5	24.6	100
≥ 2	45.8	75	37.5	25.5	84
≥ 2.5	61.1	68.8	58.9	32.3	86.8
≥ 3	62.5	43.8	67.9	28	80.9
≥ 3.5	72.2	43.8	80.4	38.9	83.3

## Discussion

The optimal management of pediatric lateral humeral condyle fractures can be challenging to extricate from the conflicting published resources. The integrity of the articular cartilaginous hinge has become a foundation for guiding management, as indicated by the recent classification systems by Weiss et al. and Song et al. [8, 9]. However, measuring displacement on radiography alone has been shown to yield inconsistent results in predicting the disruption of the articular hinge, consistent with findings from our study [6, 13, 14].

The findings of this investigation into the utility of arthrography in LHCF can therefore be summarized in three ways. Firstly, our data suggest that there are patients whose radiographs and respective degrees of displacement cannot accurately be used to determine the integrity of the articular surface. This was evident in patients that had intact articular hinges on an arthrogram after initially predicted to have been disrupted using plain radiographs (ranging 3.1–4.7 mm). The opposite was equally present, where patients had minimal initial displacement (ranging 1.5–2.6 mm) on radiographs that was subsequently confirmed as a disrupted hinge on arthrography. Thus, we deduce that a significant number of outliers to the general rule exist; those that have intact hinges above 4 mm of displacement and those with disrupted hinges below 2 mm of displacement.

Secondly, data from our study have demonstrated a modification of preoperative radiography versus intraoperative arthrography plan in 21% of the study group. Among those patients, seven of which required an open reduction after initially anticipated to require closed reduction. Whereas eight patients were managed with closed reduction and pinning and avoided open reduction after an arthrogram revealed an intact articular hinge. This is particularly advantageous in avoiding the complications associated with ORIF where CRPP can be safely performed with minimal complication rates [5, 11, 12, 15]. The opposite applies, for instance, in a patient with < 2 mm of displacement and a disrupted articular hinge requiring ORIF.

Thirdly, the accuracy of radiographs assessed in our analysis has demonstrated that the accuracy of predicting articular disruption is reduced as displacement decreases. While the classical 2 mm displacement cutoff has been utilized, patients with < 2 mm displacement and disrupted articular hinges were identified in our study group. Perhaps, a lower threshold of displacement should be considered, particularly given the higher complication rate in delayed management after initial conservative management. The latter, which often includes ORIF after non-operative failure, carries higher risks of complications including; nonunion, malunion, angular deformities [5, 12, 16, 17]. The lower threshold toward early operative management has been similarly been advocated for in a recent study by Edmonds et al., denoting a significantly higher risk of non-operative failure for fractures with a displacement of > 1.2 mm [12]. In addition, they report a 37% rate of revision treatment (late displacement) in the non-operative group. Whereas 12% of the operative group had a postoperative complication requiring treatment [12]. The early operative treatment approach may also be of particular benefit to patients that are unlikely to adhere to the appropriate follow-up. Furthermore, providers may benefit from establishing realistic expectations with patients and parents, as management is dynamic and complication rates can be high in markedly displaced fractures [18].

This diagnostic dilemma has led to the use of radiographic views, computed tomography (CT), magnetic resonance imaging (MRI), and ultrasound scans (US) as alternatives to radiography. In a cadaveric study, Knutsen et al. concluded that true fracture displacement is likely larger than the apparent radiographic displacement, reporting discrepancies of up to 1.6–6 mm [6]. The use of the MRI and US have recently been utilized to assess articular congruence yielding promising results in relatively small sample sizes [13, 14]. Although these techniques offer a noninvasive assessment of the articular surface, their general application in everyday practice may be limited by widespread availability, large radiation doses and operator dependence.

The findings of this study would suggest that an alternative, potentially more preemptive approach to LHCF may be beneficial. While the findings argue for arthrography and early operative treatment in patients displaced more than 1 mm, the results from this study should be interpreted with caution. Furthermore, evaluation of displacement on radiographs and arthrograms is often variable and not without its challenges. The results from recent retrospective reviews suggest that treatment of minimally displaced fractures have good outcomes when treated non-operatively [19]. Furthermore, in a retrospective series, Vories et al. found no statistically significant different in whether the arthrogram changes management of LHCF, reporting a change in management in 8% of the 49 included patients [20]. Nevertheless, prospective studies analyzing the utility of arthrograms in LHCF are scarce and there is a need for comparative trials to assess benefits objectively.

Although this study was prospectively designed and included a relatively large sample size in comparison to previous literature, it was limited by the lack of a control arm. Furthermore, the inclusion of fractures displaced < 2 mm and > 4 mm has not been previously assessed in a comparison of radiography and arthrography. The latter has yielded interesting results and highlights potential exceptions to the classical cutoff values for displacement.

## Conclusion

Data from this study suggest using the degree of displacement measured on plain radiography is insufficient in predicting articular incongruence. The use of arthrography appropriately stratifies patients into open versus closed reduction. Further, we advocate for a potentially lower threshold of displacement in suspecting articular involvement. Finally, we report favorable outcomes in early operative treatment for all patients with displacement 1–5 mm. A closer investigation into the factors that predict articular disruption may be of future interest.

## Data Availability

The datasets used and/or analyzed during the current study are available from the corresponding author on reasonable request.
